# High Bulk Modulus of Ionic Liquid and Effects on Performance of Hydraulic System

**DOI:** 10.1155/2014/504762

**Published:** 2014-01-02

**Authors:** Milan Kambic, Roland Kalb, Tadej Tasner, Darko Lovrec

**Affiliations:** ^1^OLMA d.d., Poljska pot 2, 1000 Ljubljana, Slovenia; ^2^Proionic GmbH, Parkring 18, 8074 Grambach, Austria; ^3^HAWE Hidravlika d.o.o., Petrovce 225, 3301 Petrovce, Slovenia; ^4^University of Maribor, Smetanova 17, 2000 Maribor, Slovenia

## Abstract

Over recent years ionic liquids have gained in importance, causing a growing number of scientists and engineers to investigate possible applications for these liquids because of their unique physical and chemical properties. Their outstanding advantages such as nonflammable liquid within a broad liquid range, high thermal, mechanical, and chemical stabilities, low solubility for gases, attractive tribological properties (lubrication), and very low compressibility, and so forth, make them more interesting for applications in mechanical engineering, offering great potential for new innovative processes, and also as a novel hydraulic fluid. This paper focuses on the outstanding compressibility properties of ionic liquid EMIM-EtSO_4_, a very important physical chemically property when IL is used as a hydraulic fluid. This very low compressibility (respectively, very high Bulk modulus), compared to the classical hydraulic mineral oils or the non-flammable HFDU type of hydraulic fluids, opens up new possibilities regarding its usage within hydraulic systems with increased dynamics, respectively, systems' dynamic responses.

## 1. Introduction

All ionic liquids (ILs) may be viewed as a new and remarkable class of solvents or as a type of material. In most cases ionic liquids are defined as salts with melting temperatures below the boiling point of water. That is only a definition based on temperature that says little about the compositions of the materials themselves, except that they are completely ionic. In reality, most ionic liquids described in the literature are liquid at room temperature. Therefore “Molten salts” are the more common and more broadly applied term for ionic compounds in the liquid state.

The general chemical composition of ionic liquids is surprisingly consistent even though the specific compositions and the chemical and physical properties vary tremendously. Most ionic liquids have an organic cation and an inorganic, polyatomic anion. Since there are many known and potential cations and anions, the potential number of ionic liquids is huge. Therefore, discovering a new ionic liquid only is relatively easy. Much more effort must be invested into determining the physical and chemical properties of ILs and their usefulness.

The earliest material that would meet our current definition of an ionic liquid was observed in Friedel-Crafts reactions in the mid-19th century as a separate liquid phase called the “red oil.” The fact that the red oil was a salt was determined more recently, when NMR spectroscopy became a commonly available tool. Early in the 20th century, some alkyl-ammonium nitrate salts were found to be liquids. In the 1960s, John Yoke at Oregon State University reported that mixtures of copper (I) chloride and alkyl-ammonium chlorides were often liquids. This was not as simple as it might appear as several chlorocuprous anions are formed, depending on the stoichiometry of the components. In the 1970s, Jerry Atwood at the University of Alabama discovered an unusual class of liquid salts he termed “liquid clathrates.” These were composed of a salt combined with an aluminium alkyl, which then formed an inclusive compound with one or more aromatic molecules. The formula for the ionic portion is M[Al_2_(CH_3_)_6_X], where M is an inorganic or organic cation and X is a halide [1].

At the beginning of the 1980s, Seddon and coworkers began to use chlorine aluminate melts as non-aqueous polar solvents for examination of transition metal catalysis. Seddon, in particular, helped the bringing of ionic liquids to public attention. Over recent years ionic liquids have gained in importance, causing a growing number of scientists and engineers to investigate possible applications of these liquids because of their unique physical and chemical properties. Their outstanding advantages make them more interesting for applications even in chemical engineering, offering great potential for new innovative processes [2].

ILs are usually composed of organic cations, typically containing nitrogen or phosphorus, and weakly coordinated anions. Some of the more common cations are imidazolium, phosphonium, pyridium, and ammonium, whilst some common anions are BF_4_, PF_6_, CF_3_SO_3,_ or the more complex N(CF_3_SO_2_)_2_.

The possibility of tuning the chemical and physical properties by changing the anioncation combination is a great opportunity for obtaining task-specific IL.

**Figure 1:**
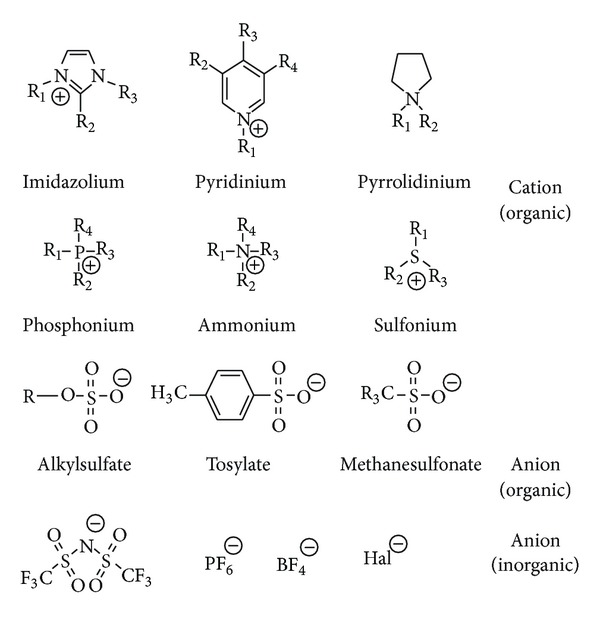
Typical structures that combine organic cations with inorganic or organic anions [3].

Today, about 1,000 ILs are described in the literature, and approximately 300 are commercially available. Typical structures that combine organic cations with inorganic or organic anions are shown in Figure 1. In the next chapters the excellent compressibility properties of the 1-ethyl-3-methylimidazolium ethylsulphate (EMIM-EtSO_4_) as an ionic liquid will be shown in comparison to the classical hydraulic mineral oil ISO VG 46, nonflammable HFD-Type hydraulic fluid Quintolubric 888-68, and water.

## 2. Compressibility of Hydraulic Fluids

The most important role of a hydraulic fluid, from amongst various roles, is the transmission of power. A bulk modulus of the fluid is crucial for hydraulic systems, particularly for high-pressure hydraulics. It can seriously affect a hydraulic system's performance in relation to position, power level, response time, and stability [4, 5]. Conventional mineral oils have rather low bulk modulus when compared to water-based hydraulic fluid. On the other hand, the water-based fluids are disadvantageous regarding lubricity and evaporation [5, 6]. So, in order to lower the power loss, shorten the response time, and increase the positional accuracy and the system's stability, the bulk modulus of oil within the hydraulic system should be improved [7].

In regard to the dynamic behavioural point of view of a hydraulic system built using classical hydraulic fluids, special attention should be paid to the compressibility of the used fluid and its impact on the specialities during the operation and performance of the hydraulic system, for example, low dynamic responses, oscillations in the motions of the hydraulic actuators, its low stiffness, low efficiencies of the actuators, and so forth. Low compressibility of a hydraulic fluid leads to the high efficiency factor of a high-pressure machine.

In general, all ILs are very incompressible media compared to the classical mineral oils—the compressibility of ILs is much lower than that of standard hydraulic mineral oil and even lower than that of water—5 × 10^−10^ Pa^−1^ [8]. This makes ILs extremely interesting for hydraulic applications such as diaphragm pumps or pulsers that determine material fatigue due to the pulsating load.

### 2.1. Methods for Determining the Compressibility of Fluids

The compressibility of fluids can be measured in two ways; through changes in pressure and the volume by a known initial volume of the fluid and through the speed of sound that spreads across the fluid. The commonly known equation for the compressibility is

(1) $$\begin{array}{c} \Delta p=-K\frac{\Delta V}{V_{0}}, \end{array}$$

where *K* is compressibility modulus [Pa], Δ*p* is change in pressure [Pa], Δ*V* is change in volume [mL], and *V*_0_ is initial volume of fluid [mL].

Equation (1) can be rewritten as

(2) $$\begin{array}{c} K=\frac{1}{\chi }=-V_{0}\frac{dp}{dV}. \end{array}$$

In order to calculate the compressibility we need to know the three variables: the volume of the fluid that we are measuring, the change in volume, and the pressure change. The volume is changed by pressing the fluid using a piston. The change in volume can be calculated from the displacement and cross-section areas of the piston (3), whilst any changes in volume caused by elongation of the measurement device must not be neglected. Consider

(3) $$\begin{array}{c} V=V_{b}-V_{\text{raz}}=\frac{S\cdot x}{1000}-V_{\text{raz}}, \end{array}$$

where *V*_*b*_ is volume, due to piston displacement [mL], *V*_raz_ is elongation volume of the measurement device [mL], *S* is cross-section area of the piston [mm^2^], and *x* is piston distance [mm].

Volume expansion for a round tube can be calculated from the deformation of the pipe's diameter under a specified pressure load (4) and the deformation of the pipe, per unit of pressure, according to (5). Only the expansion of the pipe is taken into account, whilst the expansions of the fittings are neglected during the calculation due to greater wall thickness. Consider

(4) $$\begin{array}{c} V_{\text{raz}}=\frac{\pi }{4}\left(S_{\text{DEF}}-S\right)\cdot l=\frac{\pi }{4}\left({\left(d_{n}+d_{n\text{DEF}}\cdot p\right)}^{2}-d_{n}^{2}\right)\cdot l, \end{array}$$

where *V*_raz_ is expansion volume of the measuring device [mL],  *S*_DEF_ is cross-section area of the deformed pipe [mm^2^],  *S* is cross-section area of the pipe [mm^2^],  *d*_*n*_ is inner diameter of the pipe [mm],  *d*_*n*DEF_ is diameter deformation per unit of pressure [mm/bar], *p* is pressure in pipe [bar], and *l* is pipe length [mm]. Consider

(5) $$\begin{array}{c} d_{n\text{DEF}}=\frac{d_{n}^{2}}{2\cdot E_{\text{st}}\cdot t}, \end{array}$$

where  *d*_*n*_ is inner diameter of the pipe [mm],  *d*_*n*DEF_ is diameter deformation per unit of pressure [mm/bar],  *E*_st_ is elastic modulus of the pipe [bar], and  *t* is thickness of the pipe wall [mm].

Any change in pressure can be measured using a manometer, whilst the initial volume is calculated by the weight of the liquid poured into the measuring device (6). Consider

(6) $$\begin{array}{c} V_{0}=\frac{m}{\rho }=\frac{m_{b}-m_{a}}{\rho }, \end{array}$$

where  *V* is volume of the fluid poured into measuring device [mL],  *m* is mass of the fluid poured into measuring device [g],  *m*_*b*_ is mass of the fluid and all the containers, glassware,…before pouring [g],  *m*_*a*_ is mass of the fluid and all the containers, glassware,…after pouring [g], and *ρ* is density of the fluid [g/mL].

#### 2.1.1. Measuring the Compressibility of Fluids through the Speed of Sound That Spreads across the Fluid

Sound is an example of a longitudinal wave where the waves are transmitted by means of pressure differences—compressions and rarefactions. Waves are generated by transferring the oscillation from an element (speaker membrane) to the media. It can also be generated by a single rapid movement of the membrane with large amplitude—in the air this is heard as a bang, whilst in the fluid we call this a (pressure) shock wave.

The speed of sound is dependent on the medium through which the sound is spread. The more solidly the molecules are connected and the closer they are, the faster the sound spreads. The speed of sound travelling through the fluid can be calculated according to

(7) $$\begin{array}{c} c=\sqrt{\frac{K}{\rho }}=\sqrt{\frac{1}{\chi \cdot \rho }}, \end{array}$$

where  *c* is speed of sound [m/s],  *K* is bulk modulus [Pa],  *χ* is compressibility [Pa^−1^], and  *ρ* is fluid density [kg/m^3^].

From (7), the compressibility can be expressed as

(8) $$\begin{array}{c} \chi =\frac{1}{c^{2}\rho }, \end{array}$$

where  *c* is speed of sound [m/s],  *χ* is compressibility [Pa^−1^], and  *ρ* is fluid density [kg/m^3^].

Notice that the speed of sound in materials depends only on their material properties. In the case of a conventional mineral hydraulic oil these are the bulk modulus (1, 6 GPa) and density (900 kg/m^3^), which result in approximately 1300 m/s or 4800 km/h. This means that the shock wave travels a distance of one metre in less than 1 ms.

The speed of sound can be measured in two ways:

- the change in position over a specified time period,
- the frequency of a standing wave that is formed in a two-side closed pipe.

#### 2.1.2. Measuring the Speed of Sound as a Change in Position over a Specified Time

The speed is more easily measured over a time period. It is calculated as the ratio between the distance travelled and the time spent on this.

(9) $$\begin{array}{c} c=\frac{\Delta l}{\Delta t}, \end{array}$$

where  *c* is speed of sound [m/s],  Δ*l* is distance, travelled by the sound [m], and  Δ*t* is time that sound spends to travel the distance Δ*l* [s].

Therefore, to measure the speed of a spreading shock-wave, we need two sensors, one at the beginning and the other at the end of the pipe. The sensors must be sufficiently quick and accurate to detect the shock wave front line at all. We also need a sufficiently good measuring device, which will be able to acquire the signals coming from the sensors fast enough, over very short sample times.

#### 2.1.3. Measuring the Speed of Sound through the Frequencies of Standing Waves

**Figure 2:**
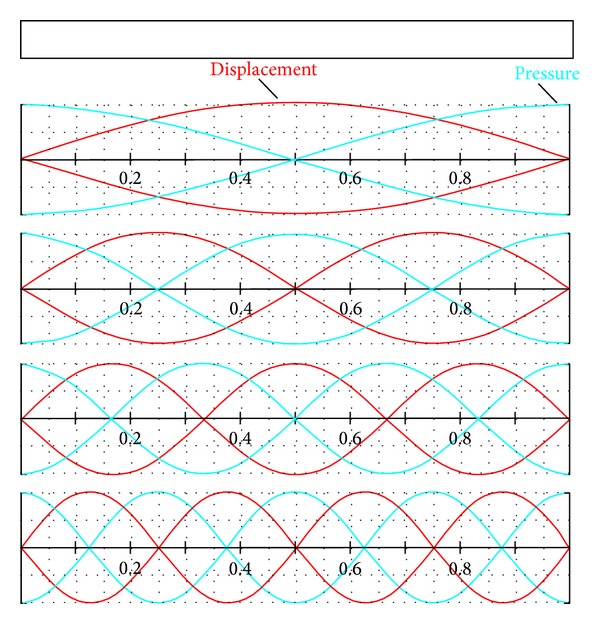
Standing wave in a pipe closed on both sides.

A standing wave is created as the sound travels through a pipe that is closed on both sides (Figure 2). Two nodes are formed at both ends of the pipe where the displacement amplitude is minimal. In case of fundamental wavelength there is an antinode in the middle of the pipe length where the displacement amplitude is maximal. In this case the wavelength of the wave equals twice the length of the tube. Higher harmonic components are also formed in the pipe and they have *n* antinodes (1, 2, 3,…), and their wavelengths equal (2/*n*) · *l*.

If we place a pressure sensor near any part of the pipe's edge, we can detect the waves as the pressure changes. The wave-motion there is caused by the pressure oscillation at a certain frequency. The frequency of the signal can be extrapolated using the so-called (FFT) analysis Fast Fourier Transformation. The speed of a standing wave is given by

(10) $$\begin{array}{c} c=\nu \cdot \lambda , \end{array}$$

where  *c* is speed of the standing wave [m/s],  *ν* is frequency of the standing wave [Hz], and  *λ* is wavelength of the standing wave [m].

For a fundamental harmonic that has maximal amplitude and its wavelength equals twice the pipe's length, we can therefore write:

(11) $$\begin{array}{c} c=\nu \cdot 2l, \end{array}$$

where *c* is speed of the standing wave [m/s],  *ν* is frequency of the standing wave [Hz], and  *l* is distance between pipe ends [m].

### 2.2. Compressibility Measuring Device

**Figure 3:**
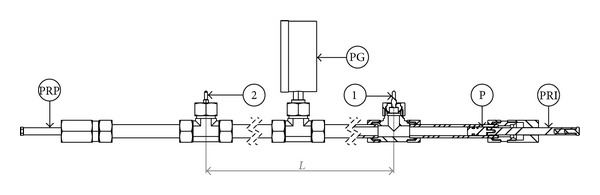
Compressibility measuring device.

The compressibility measuring device (Figure 3) was developed in such a way that the compressibility can be measured using all three ways at the same time. It consists of hydraulic tubes made of E235 steel, with size 30 × 5—outer diameter 30 mm and wall thickness 5 mm. Inside a tube the diameter and piston diameter therefore equals 20 mm. The tubes are interconnected using hydraulic fittings, heavy construction, and with a size of 30 S, which are sealed with progressive steel rings. There are pistons at each side of the device. The left piston, which is moved using a piston rod PRP, is used for squeezing the fluid. It is moved by rotating the piston rod that has an outer thread of M16 × 1.5. Fluid shrink can be calculated using a number of turns of the piston rod. When the fluid compresses, the pressure increase appears on a digital pressure gauge (PG). The right piston (P), which is moved with piston rod (PRI), is used for shock-wave generation. The difference between PRP and PRI is that PRP is threaded and PRI is only guided. A shock wave is formed by the impact on PRI in the axial direction. The shock wave is then detected by a piezoelectric pressure sensor (1) and a few milliseconds later by a second sensor (2). Both sensors are approximately 4 m apart. The speed of sound can be calculated from the signal delay. A standing wave is formed when the shock wave starts to bounce between both pistons. Therefore, the signal is being acquired after the impact in order to retrieve the standing wave's behaviour. The frequency of the standing wave can be calculated later using FFT.

All the important parameters of the measuring device can be found in Table 1.

The fluid is poured into the device through the pressure gauge (PG) mount, which is connected to the system using Minimess connection. During the pouring process no air must remain in the system. (The actual angle between the piezoelectric sensors (1, 2) and the pressure gauge (PG) is more than 90°—the piezoelectric sensors are almost upside-down so the air does not remain trapped beneath them.) When no air remains in the system, the pressure gauge (PG) can be mounted back and the measurement can begin.

**Figure 4:**
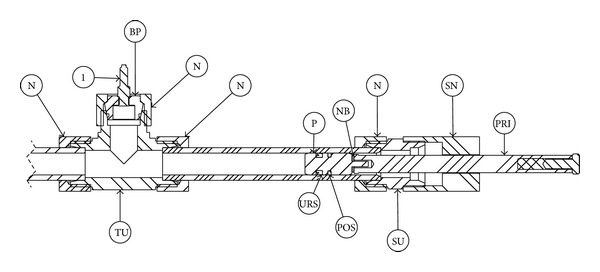
Cross-section of the compressibility measuring device.

Piezoelectric sensors (1 and 2) are mounted on the device using modified blanking plugs that have UNF 3/8′′ threads. The blanking plugs are mounted onto the fittings (TU—TEE Union, SU—Straight Union) with nuts (N), in the same way as the tubing. The piston is sealed using two seals that can withstand pressures up to 600 bars. The inner seal (URS) is a U-ring made of NBR. The outer seal (POS) consists of two parts—the PTFE profile ring that is supported by an O-ring made of NBR. In order to make sure that the piston (P) does not rotate, a needle roller bearing (NB) is placed between the piston and the piston rod (PRP, PRI). The impact piston rod (PRI) is guided by a special nut (SN) that has a clearance hole with a diameter of 17 mm. In total contrast, the nut that guides the pressurising piston rod (PRP) has an internal thread (Figure 4).

A precise electronic device KERN PCB 6000-1 with digital scale is used for weighing the used fluid. The technical data of the precise electronic device KERN PCB 6000-1 are given in Table 2.

A WIKA DG-10-S digital pressure gauge with G 1/2′′ thread is used for pressure difference measurement, which is mounted on the measuring device using Minimess coupling. This sensor has a digital display for easier reading. The technical data of the digital pressure gauge are given in Table 3.

Data acquisition is realised using a National Instruments Compact RIO controller, which is programmed within a LabVIEW programming environment. The controller has a built-in FPGA for fast real-time data processing.

The only transducer that can respond to the pressure change of a shock-wave is a piezoelectric transducer. Such a transducer utilises piezo crystal, which produces charge when deformed. The problem of such sensors is the expensive charge amplifier that is needed for amplifying the charge. Therefore, IEPE or ICP sensors have been developed. They have a MOSFET amplifier already built into the sensor.

Two Piezotronics PCB 102B sensors were chosen as the measuring devices. These sensors are supplied with constant current of 2 mA and the pressure is proportional to the voltage drop on the sensor. An IEPE module for compact RIO is used as a current supply and voltage measuring device. The technical data of the Piezotronics PCB 102B sensors are given in Table 4.

A WIKA DG-10-S digital pressure gauge with G 1/2′′ thread is used for pressure difference measurements, which is mounted on the measuring device using Minimess coupling. This sensor has a digital display for easier readings.

The compact RIO controller was chosen because of its performance, modular construction, and simple programming. The controller also has a built-in FPGA IC, which can be programmed within the same programming environment—LabVIEW.

Piezoelectric sensors are connected to IEPE module (NI9234), which is inserted into one of the controller's slots. The module is used for supplying the sensors and measuring their values. Data acquisition is performed using a program that is written on the FPGA IC. This program continuously reads both pressure signals at a sample rate of 51,2 kHz and stores 30.000 last values into a FIFO buffer.

When it detects a shock wave on the first sensor, it continues to acquire 25.000 new values. This means that we acquire 5.000 values before the shock wave and 25.000 values afterwards. Such a quantity of data can be used for accurately analysing the shock wave. Any delay between the signals on sensors 1 and 2 is calculated using a special algorithm that aligns the slopes of the first maxima of both signals. Afterwards, an FFT analysis is preformed and the frequency of the highest amplitude equals the frequency of the standing waves. The speed of sound is then calculated for both delay and frequency. The accuracy of the acquired data depends on the accuracy of the IEPE module, as shown in Table 5.

#### 2.2.1. Compressibility Measurement Procedure

The compressibility measurement was performed according to the next procedure.

1. Measuring device, fluid, and all other devices are used at room temperature (*T*).
2. Measuring device is assembled.
3. Fluid and all the equipment that comes in contact with the fluid are weighted (*m*_*b*_).
4. Fluid is poured into the device. Whilst shaking the device we make sure that there is no air left in it—we then add the fluid as necessary.
5. Remaining fluid and all the equipment that was weighted according to the third point are weighted (*m*_*a*_).
6. Measuring device is sealed.
7. The measurement during the compressing phase is conducted:
  - piston rod (PRP) is turned for 1 revolution in a clockwise direction using the HEX screw,
  - a shock-wave is produced from the impact of a hammer on the piston rod (PRI). The total number of screw revolutions (*n*), delay between signals (Δ*t*), frequency (*ν*), and pressure (*p*) is measured and recorded,
  - the above steps are repeated until a pressure of 400 bar is reached or until we run out of thread on the piston rod (PRP).
8. Measurement during the decompressing phase is conducted in the same way as previously, except that the screw is turned counter-clockwise.
9. One litre of the fluid in the device is poured into a clean plastic bottle for density analysis at different pressures within a range of 0⋯400 bar (*ρ*(*p*)).
10. The measuring device is emptied completely.
11. The measuring device and all components are dried.

#### 2.2.2. Compressibility Calculation Procedure

After the measurements, the compressibility can be calculated using all three mentioned methods in Section 2.1 calculating compressibility using change of pressure and volume, calculating compressibility using speed of sound, using change of position in time, and using standing wave frequency.

#### 2.2.3. Calculating Compressibility Using Change of Pressure and Volume

In the first step, pressure-dependent tube diameter deformation is calculated according to (5)

(12) $$\begin{array}{c} d_{n\text{DEF}}=\frac{d_{n}^{2}}{2\cdot E_{\text{st}}\cdot t}=\frac{20^{2}}{2\cdot 2,1\cdot 10^{6}\cdot 5}=1,9\cdot 10^{-5}\;\text{mm}/\text{bar}, \end{array}$$

where  *d*_*n*_ is tube inner diameter (Table 1) [mm],  *d*_*n*DEF_ is tube inner diameter increase per bar [mm/bar],  *E*_st_ is Young's modulus of the tube (Table 1) [bar], and  *t* is wall thickness (Table 1) [mm].

The filled-in volume of the tested fluid is calculated according to (6) and each point of measurement change in volume can be calculated by (13), obtained by combining (3) and (4):

(13) Vi=S·x−Vraz=π4·dn2·k1000·ni −π4((dn+dnDEF·pi)2−dn2)·lc,

where  *d*_*n*_ is piston diameter (Table 1) [mm],  *k* is thread pitch (Table 1) [mm],  *d*_*n*DEF_ is (12) [mm/bar],  *p*_*i*_ is pressure in the tube @ *i*th measurement [bar], and  *l*_*c*_ is tube length (Table 1) [m].

Finally, the compressibility can be calculated:

(14) $$\begin{array}{c} \chi_{i}=-\frac{1}{\Delta p_{i}\cdot 10^{5}}\cdot \frac{\Delta V_{i}}{V_{0}}=\frac{1}{\left(p_{i}-p_{i-1}\right)\cdot 10^{5}}\cdot \frac{\left(V_{i}-V_{i-1}\right)}{V_{0}}, \end{array}$$

where  *χ*_*i*_ is compressibility of *i*th measurement [Pa^−1^],  Δ*p*_*i*_ is change of pressure of *i*th measurement [bar],  *p*_*i*_, *p*_*i*−1_ is pressure of *i*th and of i-1th measurement [bar],  *V*_*i*_, *V*_*i*−1_ is volume of *i*th and of *i*−1th measurement (3) [mL], and  *V*_0_ is (2) [mL].

#### 2.2.4. Calculating Compressibility Using Speed of Sound

If the speed of sound is known (see (9)), compressibility can be calculated if the density is known. However, any calculation of change in density versus pressure has to be taken into account. Consider

(15) $$\begin{array}{c} \chi_{i}=\frac{1}{c_{i}^{2}\rho \left(p_{i}\right)}, \end{array}$$

where  *c*_*i*_ is speed of sound (*i*th measurement) [m/s],  *χ*_*i*_ is compressibility (*i*th measurement) [Pa^−1^], and  *ρ*(*p*_*i*_) is fluid density of *i*th measurement [kg/m^3^].

### 2.3. Calculation of Speed of Sound Using Change of Position over Time

Speed of sound can be, for our case, calculated according to (9) as

(16) $$\begin{array}{c} c_{i}=\frac{L}{\Delta t_{i}}, \end{array}$$

where  *c*_*i*_ is speed of sound (*i*th measurement) [m/s],  *L* is distance between piezoelectric sensors (Table 1) [m], and  Δ*t*_*i*_ is sound travel time between piezoelectric sensors (*i*th measurement) [s].

### 2.4. Calculation of Speed of Sound Using Standing Wave-Frequency

Speed of sound calculated according to standing wave frequency can be calculated using (11). It should be noted that the distance between the ends of the tube should be calculated, as in our case, between the pistons. The distance varies with the number of revolutions of the screw:

(17) $$\begin{array}{c} l_{i}=l_{0}-\frac{k}{1000}\cdot n_{i}, \end{array}$$

where  *l*_*i*_ is distance between pistons (*i*th measurement) [m],  *l*_0_ is distance between pistons @ 0 bar (Table 1) [m],  *k* is thread pitch (Table 1) [mm], and *n*_*i*_ is number of screw revolutions (*i*th measurement) [mm]. Consider

(18) $$\begin{array}{c} c_{i}=\nu_{i}\cdot 2l_{i}, \end{array}$$

where  *c*_*i*_ is speed of sound (*i*th measurement) [m/s],  *ν*_*i*_ is frequency of standing waves [Hz], and  *l*_*i*_ is distance between pistons (*i*th measurement) (see (17)) [m].

## 3. Measurement of Compressibility

The measurements of compressibility were conducted on ionic liquid with the chemical formula 1-ethyl-3-methylimidazolium ethyl sulphate (EMIM-EtSO_4_) in comparison with the classical mineral oil and nonflammable HFDU type of hydraulic fluid. Each fluid was compressed to 400 bar and then decompressed back to the atmospheric pressure. Data was gathered for each turn of the screw—approximately 8 bar each.

### 3.1. Calculation of Errors

In continuation the absolute errors of variable *i* will be noted as Δ_*i*_ and the relative errors as *δ*_*i*_. Absolute and relative errors of variables that were constant throughout a single measurement are shown in Table 6. Absolute and relative errors of variables that varied throughout a single measurement are shown in Table 7. Absolute and relative errors at one measuring point are shown in Table 8.

### 3.2. Results

**Figure 5:**
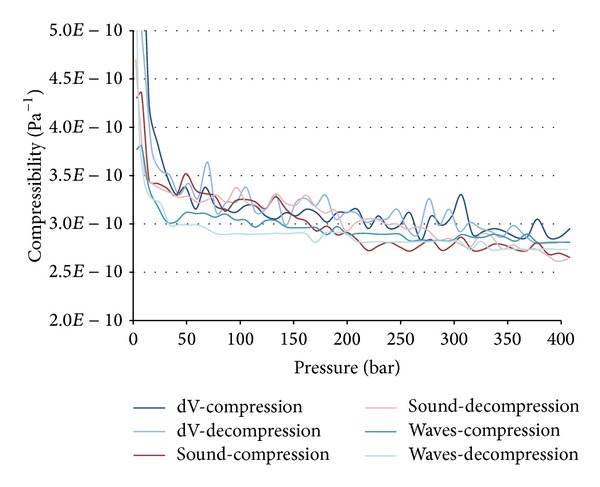
Compressibility of ionic fluid EMIM EtSO_4_.

The results from all three measurement methods during compression and decompression for ionic liquid EMIM EtSO_4_ are shown in Figure 5.

**Figure 6:**
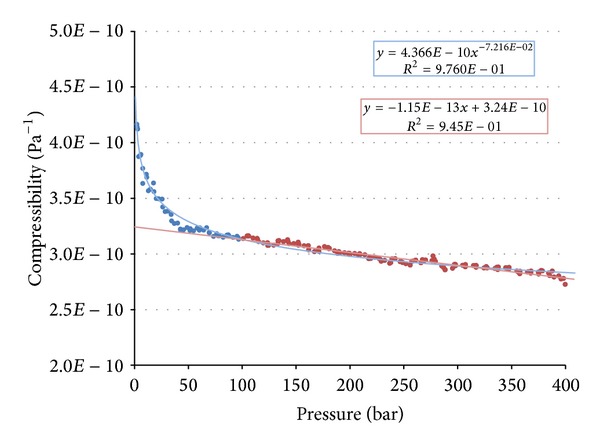
Statistically processed data.

The measured data were joined and the measurements that most likely included an error were deleted. After that a running average of 5 values was calculated. Values with a running average were approximated using exponential and linear regression. Due to influence of air dissolved in the fluid or trapped within the measuring device, only those measurements with pressure higher than 100 bars were approximated using linear regression. Both the equations and the trends are shown in Figure 6.

It can be seen that the most accurate way for estimating compressibility is linear regression and extrapolation for values lower than 100 bars. This is because the influence of air is neglected. The compressibility under atmospheric pressure can also be determined in this way.

**Figure 7:**
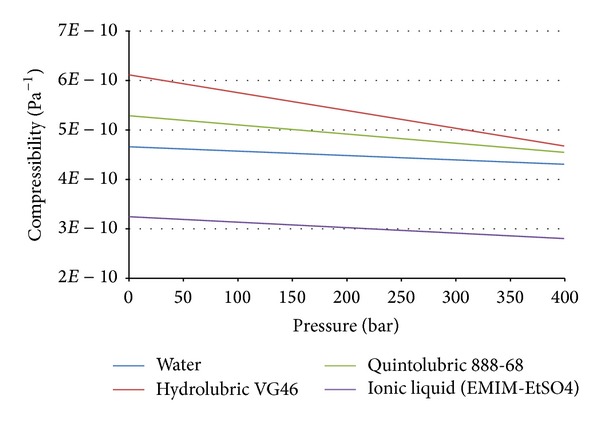
Measured compressibility versus pressure for all three considered fluids.

**Figure 8:**
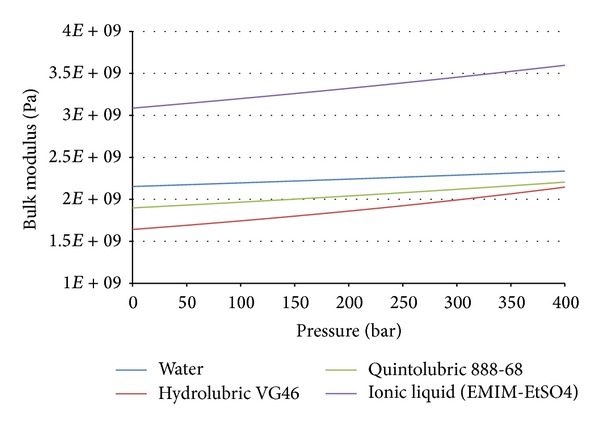
Measured bulk modulus versus pressure for all three considered fluids.

The measured compressibility can then be compared to other fluids that were measured with the same device, including the water. Additional plotted results are shown in Figures 7 and 8.

A summary of all the important chemical-physical characteristics of the tested fluids, including density, viscosity, and the viscosity index, as all being important characteristics for use as a hydraulic fluid, is shown in Table 9.

According to the data shown in Table 9, it can be concluded that the ionic liquid from this point of view has many advantages, respectively, the use as liquid with very high bulk modulus—very stiff liquid.

## 4. Benefit of Using Ionic Liquids within Hydraulic Systems

In regard to the dynamic behaviour point of view of a hydraulic system with built-in ILs as hydraulic liquid, special attention should be paid to the compressibility of the used ILs. Low compressibility of a hydraulic liquid leads to a high efficiency factor for a high-pressure machine. In general, all ILs are a very incompressible media. As shown, the compressibility of ILs is lower than that of standard hydraulic mineral oil and even lower than of water—5 × 10^−10^ Pa^−1^ [8]. This makes ILs extremely interesting for hydraulic applications such as diaphragm pumps or pulsers that determine material fatigue due to a pulsating load.

**Figure 9:**
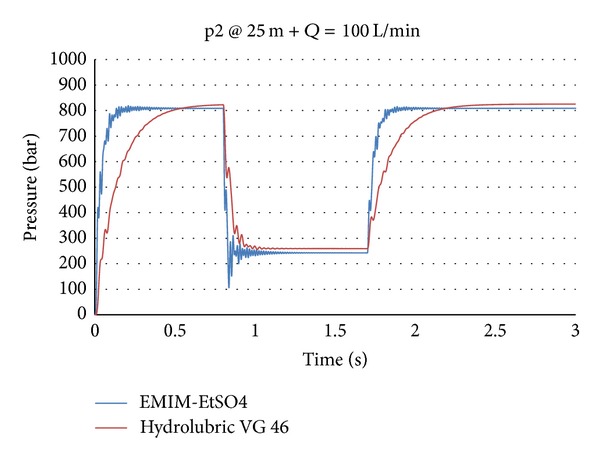
Dynamic of pressure changes by using mineral hydraulic oil and IL.

Lower compressibility enhances the efficiency factor of a machine and allows higher frequency of a hydraulic drive and the whole machine. This is because of the much higher stiffness of the used fluid and therefore the drive. As an example, Figure 9 shows the dynamic response behaviour of an initial pressure step for the case of the simplest element of a hydraulic system—dynamic of pressure changes by the opening of a directional valve at a pump flow of 100 L/min. In the shown case, a hydraulic tube of length 25 m with an inner diameter of 12 mm was used.

Figure 9 shows that the dynamic responses in the cases of using ILs as operational fluid are, by a factor of 5, higher than that in the cases of classical mineral oils. In addition, the dynamic of the entire hydraulic system is much higher. In regard to the equipment and system design point of view this means that those hydraulic components for usage within systems with ILs should be redesigned. The same applies for the whole drive system (linear or rotational drive axes) included control appliance and control concepts and controller parameter settings.

## 5. Conclusion

The presented paper focused on the usages of ILs as technical fluids. Due to their unique sets of properties unachievable by any other material, ILs open up opportunities for many different applications. They could just be a replacement for the material currently used—for example, reaction media during chemical processes. Besides being good lubricant or high-pressure hydraulic fluids, they represent an “enabling technology” that allows totally new solutions—for example, when manufacturing cellulose derivatives.

Respectively, their very high bulk modulus compared to today's usually used mineral oil offers new possibilities regarding usage within high-pressure hydraulic systems, especially within the field of application of high dynamically moved actuators.

In this work, excellent compressibility properties of the 1-ethyl-3-methylimidazolium ethylsulphate (EMIM-EtSO_4_) as an ionic liquid have been presented in comparison with the classical hydraulic mineral oil ISO VG 46, nonflammable HFD-Type hydraulic fluid Quintolubric 888-68, and water.

## Figures and Tables

**Table 1:** Important parameters of the measuring device.

Young's modulus of steel (E235 steel)	*E*_st_ = 2,1 · 10^6^ bar
Wall thickness	*t* = 5 mm
Tube inner diameter piston diameter	*d*_*n*_ = 20 mm
Thread pitch	*k* = 1,5 mm
Distance between piezoelectric sensors	*L* = 4,146 mm
Distance between pistons at 0 bar	*l*_0_ = 4,475 mm
Total length of all tubes used	*l*_*c*_ = 4,50 m

**Table 2:** KERN PCB 6000-1 scale technical data [9].

Measuring range	6000 g
Accuracy	±0,3 g
Accuracy (nonrepeatability)	±0,1 g

**Table 3:** WIKA DG-10-S digital pressure gauge technical data [10].

Pressure range	600 bar
Over pressure safety	1200 bar
Burst pressure	2400 bar
Accuracy (IEC 61298-2)	±0,5% F.S. ± 1 digit (0,1 bar) = ±3,1 bar
Accuracy (nonrepeatability)	±0,1% F.S. = ±0,6 bar

**Table 4:** Piezotronics PCB 102B pressure sensor technical data [11].

Measuring range (±5 V)	345 bar
Extended measuring range (±10 V)	690 bar
Sensitivity	0,15 mV/kPa
Resonant frequency	>500 kHz
Accuracy	<1% full scale ≤3,5 bar

**Table 5:** IEPE module (NI9234) technical data [12].

Number of channels	4
Resolution	24 bit
Sampling rate	51,2 kS/s
Accuracy	
(calibrated 25°C ± 5°C)	±0,006% F.S. = 0,3 mV
(uncalibrated 25°C ± 5°C)	±0,04% F.S. = 2,3 mV
Sample clock accuracy	±50 ppm ± 50 *μ*s/s = ±0,0512 S/s

**Table 6:** Absolute and relative errors of variables that were constant throughout a single measurement.

Variable	Source	Absolute/relative error	
*E*_st_ = 2,1 · 10^6^ bar	Subjective estimation	Δ_*E*_st__ = ±0,105 · 10^6^ bar	**δ**_**E**_st__ = 5%
*t* = 5 mm	*δ*_*d*_*n*__ = 0,75%	Δ_*t*_ = ±0,0375 mm	**δ**_**t**_ = **0,75%**
*d*_*n*_ = 20 mm	Benteler tube data	Δ_**d**_**n**__ = **±0,15 mm**	*δ*_*d*_*n*__ = 0,75%
*k* = 1,5 mm	Neglected	Δ_*k*_ = 0	*δ*_*k*_ = 0%
*L* = 4,146 m	Measuring tape accuracy	Δ_**d**_**n**__ = **±2 mm**	*δ*_*d*_*n*__ = 0,04824%
*l*_0_ = 4,475 m		Δ_**d**_**n**__ = **±2 mm**	*δ*_*d*_*n*__ = 0,04469%
*l*_*c*_ = 4,50 m		Δ_**d**_**n**__ = **±2 mm**	*δ*_*d*_*n*__ = 0,04444%

Light face cells are calculated out of bold face cells (errors from source).

**Table 7:** Absolute and relative errors of variables that varied throughout a single measurement.

Variable	Source	Absolute/relative error	
*m*_*b*_	Table 2—nonrepeatability	Δ_**m**_**b**__ = Δ_**m**_**a**__ = **±0,1 g**	Depends on variable value
*m*_*a*_			
*ρ*	Laboratory measurement	Δ_**ρ**_ = **±0,0001 ** **g/ml**	
*p*_*i*_	Table 3—accuracy	Δ_**p**_**i**__ = **±3,1 bar**	
Δ*p*_*i*_	Table 3—nonrepeatability	Δ_Δ**p**_**i**__ = **±0,6 bar**	
Δ*t*_*i*_	Table 5—sample clock accuracy	Δ_Δ**t**_**i**__ = **±0,0695 ms**	
*ν*_*i*_		Depends on the value	
*n*_*i*_	Subjective estimation	Δ_**n**_**i**__ = **±0,05**	

Light face cells are calculated out of bold face cells (errors from source).

**Table 8:** Absolute and relative errors at one measurement point.

Variable	Absolute/relative error	
*m*_*b*_ = 3515,3 g	**Table** 7	*δ*_*m*_*b*__ = ±0,002845%
*m*_*a*_ = 1654,4 g		*δ*_*m*_*a*__ = ±0,006044%
*ρ* = 1,24 g/ml		*δ*_*ρ*_ = ±0,008065%
*p*_*i*_ = 249 bar		*δ*_*p*_*i*__ = ±1,244%
Δ*p*_*i*_ = 9,9 bar		*δ*_Δ*p*_*i*__ = ±6,061%
Δ*t*_*i*_ = 2,43364 ms		*δ*_Δ*t*_*i*__ = ±2,856%
*ν*_*i*_ = 187,636 Hz	Δ_*ν*_*i*__ = ±(*ν*_*i*_ − 1/((1/*ν*_*i*_) + Δ_Δ*t*_*i*__)) = ±2,415 Hz	*δ*_*ν*_*i*__ = ±1,287%
*n*_*i*_ = 28	**Table** 7	*δ*_*n*_*i*__ = ±0,1786%
Compressibility calculated using changes of pressure and volume (Chapter 2.4.1.)		
*χ*_*i*,*dV*_ = 2,99 · 10^−10^ Pa^−1^	Δ_*χ*_*i*,*dV*__ = ±2,61 · 10^−11^	*δ*_*χ*_*i*,*dV*__ = ±8,72%
Compressibility calculated regarding change of position over time (Chapter 2.4.2.)		
*χ*_*i*,*z*_ = 2,78 · 10^−10^ Pa^−1^	Δ_*χ*_*i*,*z*__ = ±1,62 · 10^−11^	*δ*_*χ*_*i*,*z*__ = ±5,82%
Compressibility calculated using frequencies of standing waves (Chapter 2.4.2.)		
*χ*_*i*,*v*_ = 2,91 · 10^−10^ Pa^−1^	Δ_*χ*_*i*,*v*__ = ±7,80 · 10^−12^	*δ*_*χ*_*i*,*v*__ = ±2,68%
Average compressibility		
${\overset{-}{\chi }}_{i}=$2,89 · 10^−10^ Pa^−1^	$\Delta_{{\overset{-}{\chi }}_{i}}=$ ±5,21 · 10^−12^	$\delta_{{\overset{-}{\chi }}_{i}}=$ ±1,80%

Light face cells are calculated out of bold face cells (errors from source).

**Table 9:** Chemical-physical characteristics of tested fluids.

Property	Method (unit)	Sample		
		IL-EMIM-EtSO_4_	Mineral oil VG 46	Quintolubric 888-68
Density/15°C	ISO 12185 (g/cm^3^)	1,241	0,871	/
Viscosity/40°C	ASTM D 445 (mm²/s)	39,44	47,07	66,80
Viscosity /100°C	ASTM D 445 (mm²/s)	7,66	7,36	/
Viscosity index	ASTM D 2270 (−)	168	119	/
Neutralisation number	ASTM D 974 (mg KOH/g)	0,71	0,48	1,60

Compressibility	/			
0 bar	(Pa^−1^)	3,24*E* − 10	6,09*E* − 10	5,27*E* − 10
50 bar		3,18*E* − 10	5,91*E* − 10	5,18*E* − 10
100 bar		3,13*E* − 10	5,74*E* − 10	5,09*E* − 10
200 bar		3,01*E* − 10	5,38*E* − 10	4,90*E* − 10
400 bar		2,78*E* − 10	4,66*E* − 10	4,54*E* − 10

Bulk modulus	/			
0 bar	(Pa)	3,09*E* + 09	1,64*E* + 09	1,90*E* + 09
50 bar		3,14*E* + 09	1,69*E* + 09	1,93*E* + 09
100 bar		3,20*E* + 09	1,74*E* + 09	1,97*E* + 09
200 bar		3,32*E* + 09	1,86*E* + 09	2,04*E* + 09
400 bar		3,60*E* + 09	2,15*E* + 09	2,20*E* + 09
